# GBDTL2E: Predicting lncRNA-EF Associations Using Diffusion and HeteSim Features Based on a Heterogeneous Network

**DOI:** 10.3389/fgene.2020.00272

**Published:** 2020-04-15

**Authors:** Jiaqi Wang, Zhufang Kuang, Zhihao Ma, Genwei Han

**Affiliations:** School of Computer and Information Engineering, Central South University of Forestry and Technology, Changsha, China

**Keywords:** long non-coding RNA, environmental factor, heterogenous network, HeteSim score, gradient boosting decision tree, random walk with restart

## Abstract

Interactions between genetic factors and environmental factors (EFs) play an important role in many diseases. Many diseases result from the interaction between genetics and EFs. The long non-coding RNA (lncRNA) is an important non-coding RNA that regulates life processes. The ability to predict the associations between lncRNAs and EFs is of important practical significance. However, the recent methods for predicting lncRNA-EF associations rarely use the topological information of heterogenous biological networks or simply treat all objects as the same type without considering the different and subtle semantic meanings of various paths in the heterogeneous network. In order to address this issue, a method based on the Gradient Boosting Decision Tree (GBDT) to predict the association between lncRNAs and EFs (GBDTL2E) is proposed in this paper. The innovation of the GBDTL2E integrates the structural information and heterogenous networks, combines the Hetesim features and the diffusion features based on multi-feature fusion, and uses the machine learning algorithm GBDT to predict the association between lncRNAs and EFs based on heterogeneous networks. The experimental results demonstrate that the proposed algorithm achieves a high performance.

## 1. Introduction

The environment factor (EF) is a biological or non-biological factor that affects a living organism. Non-biological factors include physical factors, chemical factors, and social factors. Biological factors include parasites and viruses. Many studies have demonstrated that Gene-Environment (G–E) interactions play an important role in the etiology and progression of many complex diseases (Xu et al., 2019). Alzheimer's disease (AD), for example, is a disease that manifests as many intertwined factors, including environmental factors and the like (Eid et al., 2019). Moreover, fetal death and coronary-heart-disease (CHD) could also be caused by G–E interactions (Moreau et al., 2019).

According to the central law of molecular biology, genetic information is mainly saved in DNA sequences. Genetic information is transcribed from DNA into RNA, which is then translated into proteins. Genome sequence analysis shows that the protein-coding sequences account for about 2% of the human genome, and 98% are non-encoding protein sequences (Bertone et al., 2004). In biology, RNAs that do not code are called non-coding RNAs (ncRNAs). In ncRNAs, ncRNAs with a length between 200 and 100,000 nt are called Long non-coding RNAs (lncRNAs), and these play an important role in the understanding of life sciences (Deng et al., 2018). LncRNAs are significant in many aspects, such as in cellular biological processes, gene expression regulation at transcriptional and post-transcriptional levels, and others (Zhang Z. et al., 2019).

There are many studies on the biological mechanism and interaction between genes, microRNAs (miRNAs), lncRNAs, EFs, and diseases, such as the relationship between genes and diseases, miRNAs and diseases, lncRNAs and diseases, miRNAs and EFs, etc. Among them, microRNA (miRNA) is a kind of non-coding RNA that has only about 21–25 nucleotides (Deng et al., 2019b).

For the association between genes and diseases, a data synthesis platform based on gene variation and gene expression was established by Luo et al. This method applies the method of network analysis to predict the interaction between genes and diseases (Luo Z. et al., 2018). The recent advances in predicting gene–disease associations have been reviewed by Opap and Mulder (2017). An understanding of the association between genetics and disease is an important step in understanding the etiology of diseases. There are many other studies about the association between genes and diseases. Due to the limitation of space, only a few studies have been introduced here.

For the association between miRNAs and diseases, KBMF-MDI was proposed by Lan et al. KBMF-MDI predicts the association between miRNAs and diseases based on their similarities to diseases (Lan et al., 2018), and this is a method that is based on the dynamic neighborhood regularized logical matrix factorization (DNRLMF-MDA) proposed by Yan et al. (2017). The IMCMDA (Chen et al., 2018) was subsequently proposed by Chen et al. The IMCMDA is an inductive matrix filling model. A new computational model, called heterogeneous graph convolutional network (HGCNMDA) (Li et al., 2019), was presented by Li et al., and another method, the double Laplace regularization (DLRMC) matrix completion model, is proposed by Tang et al. (2019). Those studies have proven that the computational model could effectively predict the potential miRNA-disease associations and provide convenience for the verification experiment of biological researchers.

For the association between lncRNAs and diseases, a method to predict the association between human lncRNAs and diseases based on the random walk of the global network was proposed by Gu et al. (2017). The BRWLDA proposed by Yu et al. is a method to predict the lncRNA-disease associations based on the double random walk of heterogeneous networks (Yu et al., 2017). A global network-based framework named LncRDNetFlow (Zhang J. et al., 2019) was proposed by Zhang et al. LncRDNetFlow utilizes a flow propagation algorithm to predict lncRNA-disease associations. The calculation method LDASR was proposed by Guo et al. (2019). The LDASR analyzes the relationships between known lncRNAs and diseases to identify the relationships between lncRNAs and diseases. A bipartite graph network based on the known lncRNA-disease associations was constructed by Ping et al. (2018), and a bilateral sparse self-representation (TSSR) algorithm was proposed by Ou-Yang et al. (2019) to predict lncRNA-disease associations. A new method of lncRNA-disease-gene tripartite mapping (TPGLDA) was proposed by Ding et al. to predict the associations of lncRNA-disease, which combined the associations of gene-disease and lncRNA-disease (Ding et al., 2018). A new potential factor mixture model (LFMMs) estimation method was constructed by Caye et al. (2019), and the model is implemented in the updated version of the corresponding computer program. The ILDMSF is a novel framework that was proposed by Chen et al. (2020). Furthermore, a method named LDAH2V (Deng et al., 2019a) was proposed by Deng et al., and the HIN2Vec is used to calculate the meta-path and feature for each lncRNA-disease in the heterogeneous networks.

For the association between miRNAs and EFs, the MiREFRWR was proposed by Chen et al., and it uses the Random Walk with Restart algorithm in a complex network to predict interactions (Chen, 2016). The MEI-BRWMLL (Luo H. et al., 2018) method to reveal the relationships of miRNAs and EFs was proposed by FLuo et al. In this approach, multi-label learning and double random walk are used to predict the associations between miRNAs and EFs. These studies provide directional guidance for the analysis of complex diseases and the association between miRNAs and EFs in clinical trials (Chen et al., 2012; Qiu et al., 2012).

With the application of computing technology in the field of biology, more and more public biological databases have also been established, such as HMDD (Huang et al., 2018), miR2Disease (Jiang et al., 2008), DrugCombDB (Liu et al., 2020), and gutMDisorder (Cheng et al., 2020).

The development of genomics and bioinformatics facilitated the identification of lncRNA. LncRNA has also been found to interact with various EFs, such as chemicals, smoking, and air pollution (Flynn and Chang, 2014). It has been found that these lncRNAs and EFs may be the cause of some diseases (Chen and Yan, 2013). However, compared with protein-coding genes and miRNAs, there are fewer methods using bioinformatics and computational methods to study the association between lncRNAs and EFs, and these are also less effective. Based on the restart random walk model, the RWREFD method and a lncRNA-EF associations database, LncEnvironmentDB, were designed by Zhou et al. (2014). A method based on a binary network and resource transfer algorithm to predict the associations of lncRNA-EF was designed by Zhou and Shi (2018). The KATZ measure and Gaussian interaction profile kernel similarity are used to predict new potential associations between lncRNAs and EFs, as proposed by Vural and Kaya (2018). Three computational models for predicting the relationship between lncRNAs and EFs using the similarity of gaussian interaction properties of lncRNAs and EFs were proposed by Xu (2018). They are the prediction methods of lncRNAs and EFs association based on the Laplacian regularized least square method, the KATZ method, and the double random walk algorithm. The above studies show that the computational approach can improve the speed and reduce the cost.

However, the aforementioned studies for predicting the association between disease-related lncRNAs and EFs usually use traditional similarity search methods, which focus on measuring the similarity between objects of the same type. Those existing methods to study the association between disease-related lncRNAs and EFs simply treat all objects as the same type without considering different subtle semantic meanings of different paths in the heterogeneous network. This will reduce the accuracy and persuasiveness of the results. In this paper, we have proposed a high-performance method to predict the correlation between lncRNAs and EFs based on heterogeneous networks. The proposed method integrates the structural information and heterogenous networks and combines the Hetesim features and the diffusion features as data features and uses the GBDT algorithm as a prediction model. The HeteSim features are a path-based measurement method in heterogeneous networks and can measure the relationship between objects of the same or different types. The Hetesim has not been used to predict the association between lncRNAs and EFs. It is the first time that the Hetesim is integrated as a fusion feature in the step of feature extraction for predicting the association between lncRNAs and EFs. The method GBDT is used in the proposed algorithm, which is an integrated learning method in machine learning, and has superior accuracy compared with other algorithms. It is also the first time that the integrated learning method GBDT is used to investigate the association between lncRNAs and EFs. From our perspective, on the one hand, our proposed method provides an efficient calculation method for mining the association between lncRNAs and EFs, which greatly saves manpower and material resources. On the other hand, it also helps biologists to explore the influence of environmental factors on diseases.

For the rest of the paper, the materials and methods have been presented in section 2, the experimental results and evaluates have been discussed in section 3, and, finally, we have concluded this paper in section 4.

## 2. Materials and Methods

The data used in this experiment are downloaded from the DLREFD database (Sun et al., 2017). The data include 475 lncRNAs and 152 environmental factors. After the duplicate data are removed, the number of correlations between lncRNAs and EFs was 735. The set of lncRNAs and the set of EFs are shown in Supplementary Material.

**Figure 1:**
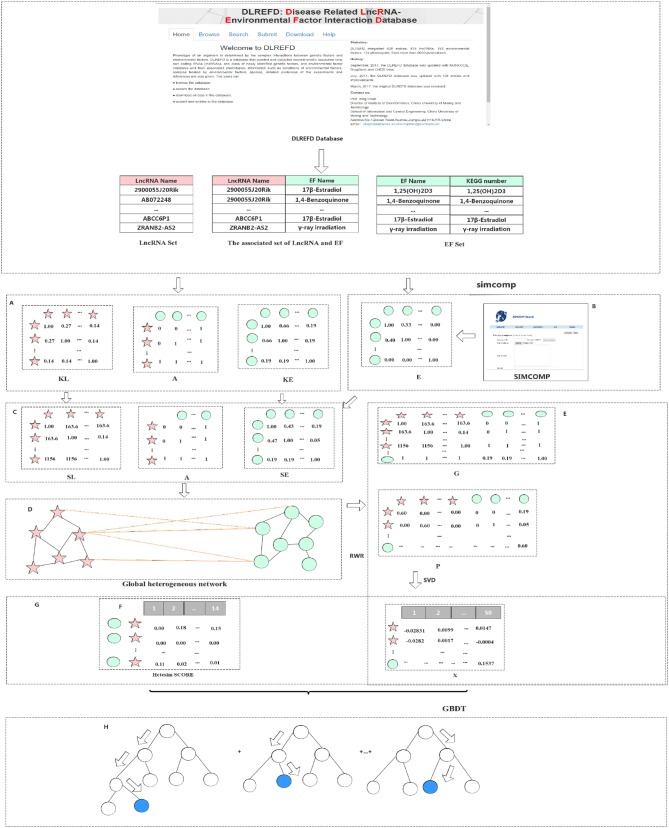
Flowchart of our method: **(A)** Obtained the association matrix A; Calculated the gaussian interaction profile kernel similarity of lncRNA and EF respectively. **(B)** Calculated the chemical structure similarity matrix E. **(C)** Obtained lncRNA similarity information SL and construct a similarity matrix SE of EF. **(D)** Integrated three subnets A, SL, and SE to construct a global heterogeneous network. **(E)** Constructed the adjacency matrix G and obtain the diffusion feature. **(F)** Calculated the Hetesim score. **(G)** Combined the diffusion feature and the HeteSim score. **(H)** Trained the Gradient Boosting Decision Tree classifier (GBDT).

A method based on the Gradient Boosting Decision Tree (GBDT) to predict the association between LncRNA and EFs (GBDTL2E) has been proposed in this section. The GDDTL2E integrates the structural information and heterogenous networks, combines the Hetesim features and the diffusion features based on multi-feature fusion, and uses the machine learning algorithm GBDT to predict the association. This mainly includes several steps: (1) according to the lncRNA-EF correlations dataset downloaded from the public database DLREFD, after the duplicate data are removed, the set of lncRNAs and EFs and the association matrix A of the lncRNA-EF correlations are obtained, respectively. Then, the gaussian interaction profile kernel similarity of lncRNA (KL) and the gaussian interaction profile kernel similarity of EFs (KE) are calculated, respectively. (2) The chemical structure similarity matrix E between EFs is calculated by using the published tool SimComp. (3) The lncRNA similar information (KL) is transformed by the logistic function to obtain lncRNA similarity information SL, and the chemical structure similarity matrix E and the gaussian interaction profile kernel similarity matrix (KE) are then used to construct a similarity matrix SE of EFs. (4) A global heterogeneous network is constructed by integrating the three subnets of association matrix A, similarity matrix SL of lncRNA, and similarity matrix SE of EFs to construct adjacency matrix G of the global heterogeneous network. On the heterogeneous network, the Random Walk with Restart (RWR) algorithm is used to calculate the diffusion score and obtain the diffusion features, and singular value decomposition (SVD) is used to reduce the dimension of the diffusion features. (5) The Hetesim feature (score) for the lncRNAs-EFs pair is calculated. (6) The feature data set is obtained by combining the diffusion feature and the HeteSim score. The obtained combined feature is used to train the Gradient Boosting Decision Tree (GBDT) for predicting the relationship between lncRNAs and EFs. Figure 1 shows that the overview of the proposed method. Each step of GBDTL2E are described in the following section.

### 2.1. Calculate Gaussian Interaction Profile Kernel Similarity

In this section, the calculation of the gaussian interaction profile kernel similarity was presented first. The association matrix A of lncRNAs and EFs was obtained by the known lncRNA-EF correlations. The gaussian interaction profile kernel similarity matrix of lncRNA and the gaussian interaction profile kernel similarity matrix of EF were calculated. Let *A*(*l*_*i*_, *e*_*j*_) indicate whether the lncRNA *l*_*i*_ is associated with *e*_*j*_. Specifically, *A*(*l*_*i*_, *e*_*j*_) = 1 if there is an association between *l*_*i*_ and *e*_*j*_; otherwise, *A*(*l*_*i*_, *e*_*j*_) = 0, which is given by

(1) $$\begin{array}{l} \text{A}\left(l_{i},e_{j}\right)=\{\begin{array}{cc} 1 & l_{i}\;is\;associated\;with\;e_{j}\\ 0 & otherwise \end{array} \end{array}$$

The gaussian interaction profile kernel similarity matrix KL of lncRNA was constructed. For a given lncRNA *l*_*i*_, *IP*(*l*_*i*_) is defined as the *i*_*th*_ row of the adjacency matrix A. Then the gaussian interaction profile kernel similarity between lncRNA *l*_*i*_ and lncRNA *l*_*j*_ for each lncRNA pair is calculated, which can be written as

(2) $$\begin{array}{l} KL\left(l_{i},l_{j}\right)=\exp\left(-\gamma_{l}\|\text{IP}\left(l_{i}\right)-\mathrm{IP}\left(l_{j}\right){\|}^{2}\right) \end{array}$$

(3) $$\begin{array}{l} \gamma_{l}={\gamma^{\prime }}_{l}/\left(\frac{1}{nl}\sum_{i=1}^{nl}\|\text{IP}\left(l_{i}\right)\right){\|}^{2}) \end{array}$$

where γ_*l*_ is used to control the frequency band of Gaussian interaction profile kernel similarity. It represents the normalized frequency band of Gaussian interaction profile kernel similarity based on the new frequency band parameter $\gamma_{l}^{\prime }$. Denote *nl* as the number of lncRNA. Denote KL as the gaussian interaction profile kernel similarity matrix of lncRNA, and denote KL(*l*_*i*_, *l*_*j*_) as the gaussian interaction profile kernel similarity score of lncRNA *l*_*i*_ and lncRNA *l*_*j*_.

Similarly, the known lncRNA-EF correlations were used to construct the gaussian interaction profile kernel similarity matrix of EFs. For a given EF *e*_*i*_, $IP^{\prime }\left(e_{i}\right)$ is defined as the *i*_*th*_ column of the adjacency matrix A. KE represents the gaussian interaction profile kernel similarity matrix of environmental factors. Denote KE(*e*_*i*_, *e*_*j*_) as the gaussian interaction profile kernel similarity score of EFs *e*_*i*_ and *e*_*j*_, which is given by

(4) $$\begin{array}{l} \text{KE}\left(e_{i},e_{j}\right)=\exp\left(-\gamma_{e}\|\text{I}{\text{P}}^{\text{′}}\left(e_{i}\right)-\text{I}{\text{P}}^{\text{′}}\left(e_{j}\right){\|}^{2}\right) \end{array}$$

(5) $$\begin{array}{l} \gamma_{e}={\gamma^{\prime }}_{e}/\left(\frac{1}{ne}\sum_{i=1}^{ne}\|{\text{IP}}^{\prime }\left(e_{i}\right)\right){\|}^{2}) \end{array}$$

where γ_*e*_ represents normalized gaussian interaction kernel similarity bandwidth based on the frequency width parameter $\gamma_{e}^{\prime }$. Denote *ne* as the number of EFs.

### 2.2. Calculate Chemical Structure Similarity

In this section, the computation of the chemical structure similarity has been given. The chemical structural similarity matrix between EFs is calculated using the SimComp tool (Hattori et al., 2010). With the Kyoto Encyclopedia of Genes and Genomes (KEGG) database entry number corresponding to EFs in the DLREFD database as the parameter, the SimComp tool is used to calculate the chemical structure similarity score. By calling SimComp's API, the chemical structure similarity score E(*e*_*i*_, *e*_*j*_) of each pair of environmental factors *e*_*i*_ and *e*_*j*_ was calculated. SimComp (Similar Compound) is a kind of method based on a graph that is used to compare the chemical structure. It has been implemented in a KEGG system to search for similar chemical structures in a chemical structure database.

### 2.3. Obtain the Similarity Matrix

The structural information and heterogenous networks were integrated in the proposed GBDTL2E. The transformed similarity matrix SL and integrated similarity matrix calculation SE have been described in this section. The lncRNA similarity matrix KL was transformed by logistic function to obtain lncRNA similar matrix SL. The similarity matrix SE of EFs was constructed by using the chemical structure similarity matrix E of EFs and the gaussian interaction profile kernel similarity matrix KE of EFs, given by

(6) $$\begin{array}{l} \mathrm{SL}\left(l_{i},l_{j}\right)=\frac{1}{1+e^{c\cdot KL\left(l_{i},l_{j}\right)}+v} \end{array}$$

where *c* = −15, *v* = log(9999);

(7) $$\begin{array}{l} \mathrm{SE}\left(e_{i},e_{j}\right)=\{\begin{array}{cc} ew\cdot E\left(e_{i},e_{j}\right)+(1-ew)\cdot KE\left(e_{i},e_{j}\right) & \text{E}\left(e_{i},e_{j}\right)\neq 0\\ KE\left(e_{i},e_{j}\right) & otherwise \end{array} \end{array}$$

where *ew* is the weight of correlation information of two EFs in SE.

### 2.4. Obtain Low-Dimensional Network Diffusion Features

In this section, the association matrix A of lncRNA-EF, the similarity matrix SL of lncRNA, and the similarity matrix SE of EFs were integrated to construct a global heterogeneous network. In heterogeneous networks, the Random Walk with Restart (RWR) is used to calculate the diffusion score and obtain the diffusion features. Due to the fact that the higher-dimensional features in model training are more susceptible to noise interference, the singular value decomposition (SVD) is used to reduce the dimension of the diffusion features. The details of each sub-steps were as follows.

#### 2.4.1. Construct of Roaming Network

In this section, the roaming network was constructed firstly. The adjacency matrix *G* of the global heterogeneous network was obtained. The matrix *G* has *nl* + *ne* dimensions, where *nl* is the number of lncRNA and *ne* is the number of EFs, respectively. G is given by

(8) $$\begin{array}{l} G=\left[\begin{array}{cc} SL & A\\ A^{T} & SE \end{array}\right] \end{array}$$

where A^T^ represents the transpose of *A*, and *SL* and *SE* are given by (6) and (7), respectively. *T* is the transition probability matrix of *G*, which is given by

(9) $$\begin{array}{l} T\left(i,j\right)=\frac{G\left(i,j\right)}{\sum_{k=1}^{nl+ne}G\left(k,j\right)} \end{array}$$

where *T*(*i, j*) represents the probability of node *i* transferring to node *j* in the global network. For any two given nodes *i* and *j* in the wandering network, if *T*(*i, j*) is not 0, there is an edge between them. If *T*(*i, j*) is 0, and node *i* has no relationship with node *j*.

#### 2.4.2. Obtain the Diffusion Features Using RWR

The RWR algorithm (Liu et al., 2016) is used to obtain the diffusion features of each node on the global network in this section. Based on the transition probability matrix *T*, the diffusion features of all nodes P = [*P*^*i*^] were obtained by RWR, where *i* ∈ {1, 2, …*n*}. *P*^*i*^ represents the diffusion features of node *i*, *n* = *nl* + *ne*, and *nl* + *ne* is the total number of nodes in the global heterogeneous network. Starting from a node *i* in the global heterogeneous networks, each step prompted two choices: randomly select the neighboring node or return the starting node. The process of restarting the random walk is given by

(10) $$\begin{array}{l} P_{t+1}^{i}=\left(1-r\right)*T*P_{t}^{i}+r*P_{0}^{i} \end{array}$$

where *r* is the restart probability; $P_{t}^{i}$ is an n-dimensional probability distribution vector of node *i*, and its *j*_*th*_ element represents the probability of accessing node *j* at step *t*, and *j* ∈ {1, 2, …, *n*}. $P_{0}^{i}$ represents the initial transition probability, which is given by

(11) $$\begin{array}{l} P_{0}^{i}=\left(\frac{1}{n},\frac{1}{n},\frac{1}{n}\ldots \frac{1}{n}\right) \end{array}$$

The initial assumption is that the transition probability value of each node is 1/*n*, and *n* is the total number of nodes. After several iterations, when (*P*_*t*+1_ − *P*_*t*_) is less than 10^−10^, the final diffusion features were obtained.

#### 2.4.3. Calculate Low-Dimensional Diffusion Features

The calculation of low-dimensional diffusion features has been given in this section following the diffusion features obtained by RWR. As the number of nodes increases, the diffusion state increases in dimension as well. Singular value decomposition (SVD) (Golub and Reinsch, 1971; Cho et al., 2015) is used to reduce the dimension of diffusion features. The high-dimensional diffusion feature matrix is decomposed:

(12) $$\begin{array}{l} P=U\Sigma V^{T} \end{array}$$

(13) $$\begin{array}{l} P=U\Sigma^{1/2}\Sigma^{1/2}V^{T} \end{array}$$

where U and V represent the left singular matrix and the right singular matrix, respectively. The U and V are units on an orthogonal matrix, Σ only has value on the diagonal, and the other elements are 0. We refer to these non-zero values as singular values and order these values in Σ from largest to smallest. Singular values can be thought of as representing values of a matrix, or as representing information about the matrix. The larger the singular value, the more information it represents. Therefore, in order to reduce the computation, we only need to take the first 50 maximum singular values, and we can basically restore the data itself. Therefore, we take the first 50 singular values and eigenvectors, which are given by

(14) $$\begin{array}{l} X=U_{n*d}{\left(\Sigma_{d*d}\right)}^{1/2} \end{array}$$

(15) $$\begin{array}{l} W={\left(\Sigma_{d*d}\right)}^{1/2}{\left(V_{d*n}\right)}^{T} \end{array}$$

where X is the low-dimensional node feature matrix derived from the high-dimensional diffusion feature. Each row of matrix X is the low-dimensional feature vector of each node in the network. W is the low-dimensional context eigenmatrix derived from the high-dimensional diffusion feature. Thus, we obtain the diffusion feature X after dimensionality reduction.

### 2.5. Calculate the Hetesim Score

**Figure 2:**
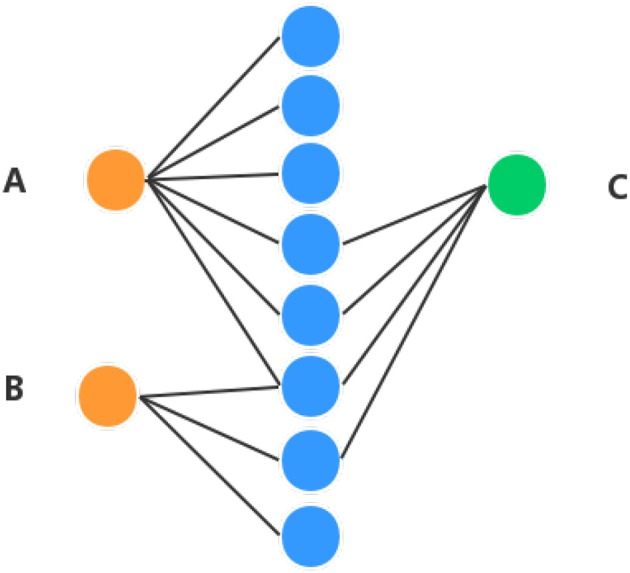
Example of understanding HeteSim masure. Different color circles denote three different kinds of objects in the heterogeneous network. **(A–C)** represent three different nodes in the heterogeneous network.

In order to obtain high performance, apart from the diffusion feature obtained in the above section, the proposed method combines the Hetesim features and the diffusion features based on multi-feature fusion. Another important feature is that HeteSim (Shi et al., 2014) is used to calculate the relevance between objects in the heterogeneous network in this section. HeteSim is a path-based measure. For each pair object (of the same or different types) in the heterogeneous network, it could obtain one single score, which means their relatedness based on an arbitrary path. Figure 2 illustrates a HeteSim score.

As we can see from Figure 2, the number of paths from A to C is three and the number of paths from B to C is two. The number of paths from A to C is larger than B to C, which might mean that A is closer to C than B. But, based on HeteSim, B is closer to C than A to C because there are two edges for B to C, which account for two-thirds of the edges starting from B to other objects. However, A only has a small part of the edges connected with C. In our proposed method, the HeteSim is used to measure the similarities between lncRNAs and EFs. Under the constraint of length less than five, there are 14 different paths from lncRNA to the EFs, as shown in Table 1.

**Table 1:** The paths from a lncRNA to an environmental factor in our heterogeneous network with a length of less than 5.

**Id**	**Path**	**Meaning**	**Length**
1	LLE	lncRNA-lncRNA-EF	2
2	LEE	lncRNA-EF-EF	2
3	LLLE	lncRNA-lncRNA-lncRNA-EF	3
4	LELE	lncRNA-EF-lncRNA-EF	3
5	LLEE	lncRNA-lncRNA-EF-EF	3
6	LEEE	lncRNA- EF-EF-EF	3
7	LLLLE	lncRNA-lncRNA-lncRNA-lncRNA-EF	4
8	LLLEE	lncRNA-lncRNA-lncRNA-EF-EF	4
9	LLELE	lncRNA-lncRNA-EF-lncRNA-EF	4
10	LLEEE	lncRNA-lncRNA-EF-EF-EF	4
11	LELLE	lncRNA-EF-lncRNA-lncRNA-EF	4
12	LELEE	lncRNA-EF-lncRNA- EF-EF	4
13	LEELE	lncRNA-EF-EF-lncRNA-EF	4
14	LEEEE	lncRNA-EF-EF-EF-EF	4

The HeteSim score between lncRNA and EF is calculated:

**Step (1):** The transition probability matrix *M*_*LP*_ from lncRNA to EF, lncRNA to lncRNA, EF to lncRNA, and EF to EF in global heterogeneous networks are calculated. The calculation formula of transfer probability matrix *M*_*LP*_(*i, j*) is given by
  (16) $$\begin{array}{l} M_{LP}\left(i,j\right)=\frac{I_{LP}\left(i,j\right)}{\sum_{k=1}I_{LP}\left(i,k\right)} \end{array}$$
  where *L* and *P* represent two types of objects in the global heterogeneous network, and *i* and *j* represent two nodes in the global heterogeneous network. Matrix *I* is the incidence matrix of *L* and *P*. If both *L* and *P* are environmental factors, matrix *I* is matrix SE. If both *L* and *P* are lncRNAs, matrix *I* is matrix SL. If *L* and *P* are lncRNA and EFs respectively, then matrix *I* is matrix A. The four transfer probability matrices can be obtained as *M*_*LE*_, *M*_*LL*_, *M*_*EL*_, and *M*_*EE*_ respectively.
**Step (2):** The *path* = (*h*_1_, *h*_2_, ⋯ , *h*_*m*+1_) is divided into two parts. When the path length *m* is even, divide the path into *path*_*L*_ = (*h*_1_, *h*_2_, ⋯ , *h*_mid_) and *path*_*R*_ = (*h*_*mid*_, *h*_2_, ⋯*h*_*m*+1_), *mid* = (*m*/2) + 1; Otherwise, when the length of path *m* is odd, we need to take *mid* = ((*m* + 1)/2) and *mid* = ((*m* + 3)/2), respectively. Then, we can get different HeteSim scores when taking the two *mid*, and the final score is the average of the two HeteSim scores.
**Step (3):** The reachable probability matrix *R*_*path*_ under *path*_*L*_ and *path*_*R*_ is calculated. The reachable probability matrix *R*_*pat*_*h*__*L*__ and *R*_*pat*_*h*__*L*__ are given by
  (17) $$\begin{array}{l} R_{path_{L}}=M_{h_{1},h_{2}},M_{h_{2},h_{3}}\cdots M_{h_{mid-1},h_{mid}} \end{array}$$
  (18) $$\begin{array}{l} R_{path_{R}}=M_{h_{mid},h_{mid+1}},M_{h_{mid+2},h_{,id+3}}\cdots M_{h_{m-1},h_{m}} \end{array}$$
**Step (4):** The HeteSim score of path *path* is calculated, which is given by:
  (19) $$\begin{array}{l} \text{Hetesim}=\frac{R_{{path}_{L}}{\left(R_{{path}_{R}^{-1}}\right)}^{T}}{\|R_{{path}_{L}}{\|}_{2}*\|R_{{path}_{R}^{-1}}{\|}_{2}} \end{array}$$
  where ${path}_{R}^{-1}$ is the reverse path of *path*_*R*_. There are in total 14 different paths from a lncRNA to an EF under the constraint of length <5. So, we obtain 14-dimensional HeteSim features for each node in the heterogeneous networks.

### 2.6. Train the Gradient-Boosting Decision Tree Classifier

After the multi-features were combined, the Hetesim features and the diffusion features were obtained. The method for training the GBDT classifier model to predict the association between lncRNAs and EFs based on heterogeneous networks has been presented in this section. The 50-dimensional diffusion features and 14-dimensional HeteSim scores were combined to get the 64-dimensional features data set. The features of the data were used for training the Gradient Boosting Decision Tree (GBDT) (Friedman, 2001) classifier. The classifier was used to predict the correlation between lncRNAs and EFs.

GBDT is an effective machine learning method for classification and regression problems. GBDT is composed of multiple decision trees, and the final answer is obtained via the sum of the conclusion of all trees. GBDT generates a weak classifier in each iteration through multiple rounds of iteration. Each classifier is trained on the basis of the gradient (residual value) of the previous round of classifiers. The final total classifier is obtained by weighted summation of the weak classifier obtained in each round of training, which is the addition model. The model training steps have been presented:

**Step (1):** The initialization model is given by:
  (20) $$\begin{array}{l} \Theta_{0}\left(x\right)=\frac{1}{2}*\log\left(\frac{\sum_{i=1}^{N}y_{i}}{\sum_{i=1}^{N}1-y_{i}}\right) \end{array}$$
  where *N* is the number of training samples, and *y*_*i*_ is the real label. The loss function is given by:
  (21) $$\begin{array}{l} L\left(\text{y},\Theta_{m-1}\left(x_{i}\right)\right)=\log\left(1+\exp\left(-\text{y}\Theta_{m-1}\left(x_{i}\right)\right)\right) \end{array}$$
  where *y* is the real class label, and Θ_*m*_(*x*) is the weak model in the *m*_*th*_ round.
**Step (2):** Cycle m in turn, where m = 1,2,…M
  **A:** The calculation for the negative gradient of the loss function of the *i*_*th*_ sample in the *m*_*th*_ round is given by:
  (22) $$\begin{array}{l} r_{m,i}=-\frac{\partial \text{L}\left({\text{y}}_{i},\Theta_{m-1}\left(x_{i}\right)\right)}{\partial \Theta_{m-1}\left(x_{i}\right)}=\frac{{\text{y}}_{i}}{\left(1+\exp\left({\text{y}}_{i}\right)\text{Θ}\left(x_{i}\right)\right)} \end{array}$$
  where *i* = 1, 2, …*N*.
  **B:** Construct the *m*_*th*_ decision tree, and then get the corresponding leaf node area *R*_*m, j*_, *wherej* = 1, 2, …, *J*, and the *J* is the number of leaf nodes in the tree.
  **C:** For the samples in each leaf node, we calculated the *c*_*m, j*_, which minimizes the loss function, namely, the best output value of fitting the leaf node, given by:
  (23) $$\begin{array}{l} c_{m,j}=\arg\underset{c}{\min}\underset{x\in R_{m,j}}{\sum }\log\left(1+\exp\left(-{\text{y}}_{i}\Theta \left(x_{i}\right)+\text{c}\right)\right) \end{array}$$
  **D:** Update *m*_*th*_ weak model:
  (24) $$\begin{array}{l} \Theta_{m}\left(x\right)=\Theta_{m-1}\left(x\right)+lr*\overset{J}{\underset{j=1}{\sum }}c_{m,j}I\left(x\in R_{m,j}\right) \end{array}$$
  where *I*(*x* ∈ *R*_*m, j*_) means that if *x* falls on a leaf node corresponding to *R*_*m, j*_, then the corresponding term is 1, and *lr* means learning rate.
  **E:** Judge whether m is greater than M. If m is less than M, then m=m+1 and jump to Step(1) for the next iterations. Otherwise, it means that m weak learners have been constructed, and we then jump to Step(3) to end the training.
**Step (3):** Obtain the final Strong Model:
  (25) $$\begin{array}{l} \Theta \left(x\right)=\Theta_{0}\left(x\right)+lr*\overset{M}{\underset{m=1}{\sum }}\overset{J}{\underset{j=1}{\sum }}c_{m,j}I\left(x\in R_{m,j}\right) \end{array}$$

### 2.7. GBDTL2E Algorithm

In this section, the proposed GBDTL2E algorithm to predict the association between lncRNAs and EFs based on heterogeneous networks has been described in Algorithm 1. From lines four to nine of Algorithm 1, the low-dimensional diffusion feature matrix X was obtained by using the random walk with restart algorithm and singular value decomposition. In lines 10–41 of Algorithm 1, the Hetesim score was obtained. In lines 42–58 of Algorithm 1, the training data is obtained and used to train the GBDT classifier. Furthermore, the final classification model is obtained.

**Algorithm 1:** GBDTL2E algorithm

**Input:** lncRNAs set, EFs set, The association matrix of the lncRNA-EFs *A*;
**Output:** The gaussian interaction profile kernel similarity matrices *KL* and *KE*. The chemical structural similarity matrix, *E*. The similarity matrices *SL* and *SE*.
1: Construct the adjacency matrix *G*;
2: Initialize the global transition probability matrix *T*;
3: Initialize the transition probability vector for each node $P_{0}^{i}=\left(\frac{1}{n},\frac{1}{n},\frac{1}{n}\ldots \frac{1}{n}\right)$
4: **while** $P_{t+1}^{i}-P_{t}^{i}>10^{-10}$ **do**:
5: Obtain the updated probability vector:
6: $P_{t+1}^{i}=\left(1-r\right)*T*P_{t}^{i}+r*P_{0}^{i}$;
7: **end while**
8: $\text{P}=U_{n*d}\Sigma_{d*d}V_{d*n}^{T}$
9: $X=U_{n*d}\Sigma_{d*d}^{1/2}$
10: Input L,P to caculate *M*_*LP*_(*i, j*)
11: **if** *L* ∈ *EFs* and *P* ∈ *EFs* **then**
12: *M*_*LP*_(*i, j*)= $M_{EE}\left(i,j\right)=\frac{SE_{EE}\left(i,j\right)}{\underset{k=1}{\sum }SE_{EE}\left(i,k\right)}$
13: **end if**
14: **if** *L* ∈ *lncRNAs* and *P* ∈ *EFs* **then**
15: *M*_*LP*_(*i, j*)= $M_{LE}\left(i,j\right)=\frac{A_{LE}\left(i,j\right)}{\underset{k=1}{\sum }A_{LE}\left(i,k\right)}$
16: **end if**
17: **if** *L* ∈ *EFs* and *P* ∈ *lncRNAs* **then**
18: *M*_*LP*_(*i, j*)= $M_{EL}\left(i,j\right)=\frac{A_{EL}^{T}\left(i,j\right)}{\underset{k=1}{\sum }A_{EL}^{T}\left(i,k\right)}$
19: **end if**
20: **if** *L* ∈ *lncRNAs* and *P* ∈ *lncRNAs* **then**
21: *M*_*LP*_(*i, j*)= $M_{LL}\left(i,j\right)=\frac{SL_{LL}\left(i,j\right)}{\underset{k=1}{\sum }SL_{LL}\left(i,k\right)}$
22: **end if**
23: **for** *n* = 1 → 5 **do**
24: Divide the path into two parts.
25: **if** *n%*2 == 0 **then**
26: *mid* = (*m*/2)+1
27: *path*_*L*_ = (*h*_1_, *h*_2_, ⋯ , *h*_mid_)
28: *path*_*R*_ = (*h*_*mid*_, *h*_2_, ⋯*h*_*m*+1_)
29: **end if**
30: **if** *n%*2! = 0 **then**
31: *mid*1 = ((*m*+1)/2)
32: *mid*2 = ((*m*+3)/2)
33: *path*_*L*_1__ = (*h*_1_, *h*_2_, ⋯ , *h*_mid1_)
34: *path*_*R*_1__ = (*h*_*mid*1+1_, *h*_2_, ⋯*h*_*m*+1_)
35: *path*_*L*_2__ = (*h*_1_, ⋯ , *h*_mid2_)
36: *path*_*R*_2__ = (*h*_*mid*2+1_, ⋯*h*_*m*+1_)
37: **end if**
38: *R*_*pat*_*h*__*L*__ = *M*_*h*_1_, *h*_2__, *M*_*h*_2_, *h*_3__⋯*M*_*h*_*mid*−1_, *h*_*mid*__
39: *R*_*pat*_*h*__*L*__ = *M*_*h*_1_, *h*_2__, *M*_*h*_2_, *h*_3__⋯*M*_*h*_*mid*−1_, *h*_*mid*__
40: $\text{Hetesim}=\frac{R_{{path}_{L}}{\left(R_{{path}_{R}^{-1}}\right)}^{T}}{\|R_{{path}_{L}}{\|}_{2}*\|R_{{path}_{R}^{-1}}{\|}_{2}}$
41: **end for**
42: Combined with the diffusion feature and HeteSim score to get the data set
43: D_train_ = {(*x*_1_, *y*_1_), (*x*_2_, *y*_2_), …, (*x*_*N*_, *y*_*N*_)}, D_test_ = {(*x*_1_, *y*_1_), (*x*_2_, *y*_2_), …, (*x*_*N*_, *y*_*N*_)}
44: Use D_train_ to train the Gradient Boosting Decision Tree (GBDT).
45: Initialize the model as Θ_0_(*x*)
46: **for** *m* = 1 → *M* **do**
47: **for** *i* = 1 → *N* **do**
48: Calculate loss function: L(y, Θ_*m*−1_(*x*_*i*_))
49: Calculate the residuals: *r*_*m, i*_
50: **end for**
51: Construct the *m*_*th*_ decision tree,
52: Get the corresponding leaf node area *R*_*m, j*_, *j* = 1, 2, …, *J*
53: **for** *J* = 1 → *J* **do**
54: Calculate *c*_*m, j*_
55: **end for**
56: Update weak model: Θ_*m*_(*x*)
57: **end for**
58: Get the strong model Θ_*M*_(*x*)

## 3. Result and Discussion

### 3.1. Data Sets

We randomly selected 300 positive samples and 300 negative samples for training the model. Positive samples were that samples with a correlation between lncRNA and EF, while negative samples were samples without a correlation between lncRNA and EF. For objective performance evaluation, an independent test set was built by randomly selecting 300 positive samples and 300 negative samples. Note that all the positive and negative samples in these test sets were independently chosen and excluded from the training set.

### 3.2. Performance Measures

The 10-fold cross-validation was used to measure the performance of the GBDTL2E. The GBDTL2E parameters used are listed in Table 2. The detailed process of 10-fold cross-validation has been described as: the training set was randomly divided into 10 groups of roughly the same size subsets. Each subset was used for validation data in turn, and the remaining nine subsets were used for training data. This process was repeated 10 times, and performance assessments were performed using average performance measures of more than 10 times. The experiment used a variety of methods to evaluate performance, including recall (REC), F1-score, accuracy (ACC), Matthews correlation coefficient (MCC), and the area under the receiver operating characteristic curves (AUC). They were defined:

(26) $$\begin{array}{l} Recall=\frac{TP}{TP+FN}, \end{array}$$

(27) $$\begin{array}{l} Accuracy=\frac{TP+TN}{TP+TN+FP+FN}, \end{array}$$

(28) $$\begin{array}{l} F1-Score=\frac{2\times TP}{2TP+FP+FN}, \end{array}$$

(29) $$\begin{array}{l} MCC=\frac{TP\times TN-FP\times FN}{\sqrt{\left(TP+FP\right)\left(TP+FN\right)\left(TN+FP\right)\left(TN+FN\right)}} \end{array}$$

where *TP* and *FP* represent the numbers of correctly predicted positive and negative samples, and *FP* and *FN* represent the numbers of wrong predicted positive and negative samples, respectively. The AUC score is computed by varying the cutoff of the predicted scores from the smallest to the greatest value.

**Table 2:** The experimental parameters of GBDTL2E.

**Notation**	**Value**	**Definition**
*nl*	475	The number of lncRNAs
*ne*	152	The number of EFs
*n*	627	The sum number of EFs and lncRNAs
$\gamma_{l}^{\prime }$	1	The frequency band of gaussian interaction profile kernel similarity of lncRNA
$\gamma_{e}^{\prime }$	1	The frequency band of gaussian interaction profile kernel similarity of EF
*ew*	0.7	The weight parameter of correlation information of two environmental factors in SE
*m*	5	The length constraint in Hetesim
*d*	50	The dimension of the low-dimensional diffusion features
*r*	0.5	The restart probability in the random walk with restart
*N*	600	The number of training samples
*M*	10	The number of training iterations

### 3.3. Performance Comparison With Existing Machine Learning Methods

**Figure 3:**
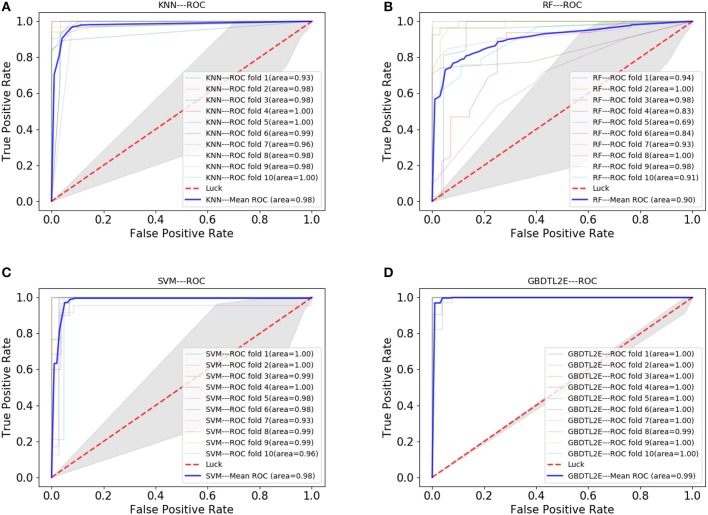
The ROC curve comparison with other machine learning methods. **(A)** The ROC curve with using KNN. **(B)** The ROC curve with using RF. **(C)** The ROC curve with using SVM. **(D)** The ROC curve with using GBDT.

**Figure 4:**
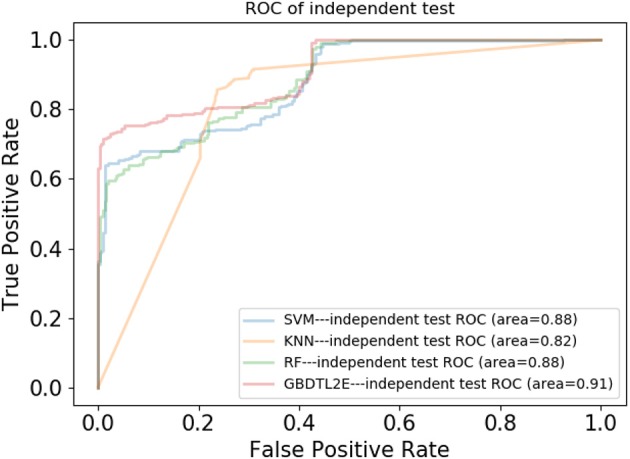
The ROC curves comparison with other machine learning methods on independent dataset.

In this section, the proposed GBDTL2E method was compared with the following schemes, which include the k-nearest neighbor algorithm (KNN) (Cover and Hart, 1967), random forest (RF) (Liaw et al., 2002), and support vector machine (SVM) (Burges, 1998). The 10-fold cross-validation was used by the four algorithms. For the KNN classifier, five nearest neighbors were used. The RF algorithm constructed multiple decision tree classifiers for training on a set of randomly selected benchmark samples to improve performance. For the SVM, we used the radial basis function (RBF) as the kernel function to optimize the penalty *c* and γ parameters. In addition, we set *c* and γ as 64 and 0.0001, respectively. Table 3 and Figure 3 show the predictive performance comparison of the machine learning approach used with other machine learning approaches. It can be seen that the method used in the present invention had the best performance. In order to further prove the performance of this model, we also compared the performances of these different machine learning methods on the independent test set. The ROC curve compared on the independent test set is shown in Figure 4. The AUC of GBDTL2E, KNN, RF, and SVM were 0.91, 0.82, 0.88, and 0.88, respectively. The results show that the performance using GBDT was better than that of other machine learning methods.

**Table 3:** The performance comparison with other machine learning methods.

**Method**	**ACC**	**RECALL**	**F1-score**	**MCC**	**AUC**
KNN	0.953	0.937	0.952	0.907	0.985
RF	0.863	0.827	0.849	0.739	0.912
SVM	0.966	0.967	0.966	0.933	0.988
GBDTL2E	0.975	0.967	0.976	0.949	0.997

### 3.4. Performance Comparison With Different Topological Features

**Figure 5:**
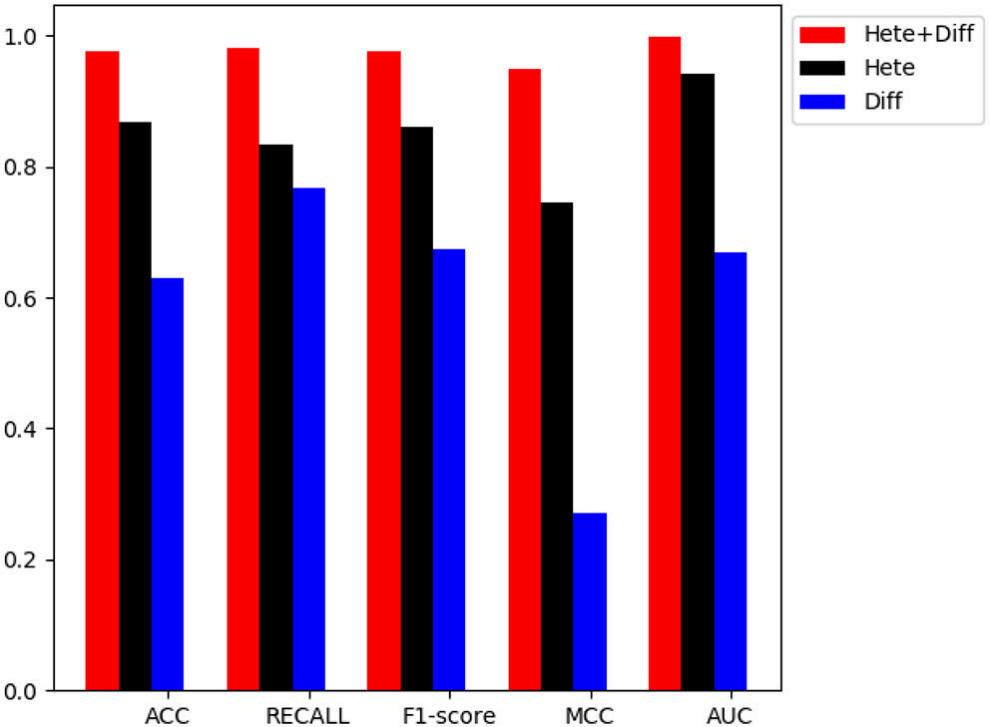
The performance comparison of different feature groups (Diffusion, HeteSim and combined feature).

**Figure 6:**
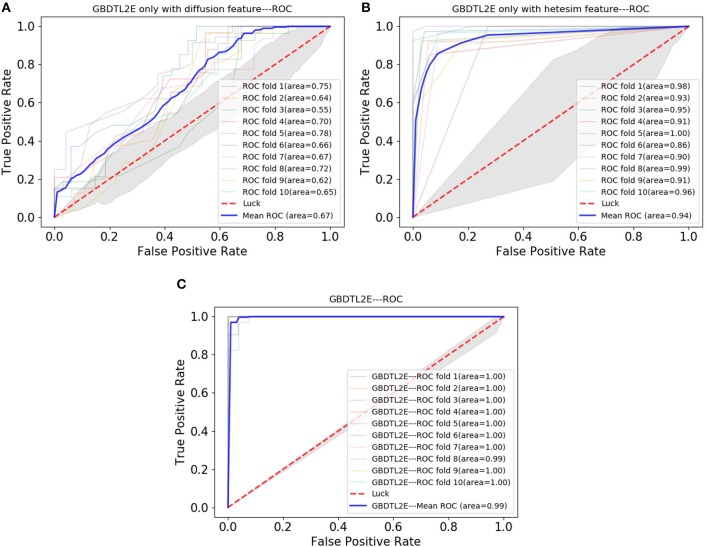
The ROC curve comparison with different feature groups. **(A)** The ROC curve only with diffusion feature. **(B)** The ROC curve only with HeteSim feature. **(C)** The ROC curve with combined feature.

In order to verify the performance of combined diffusion and Hetesim features in GBDTL2E, we compared the performance by using two separate features and combined features in this section. Figures 5, 6 show the Performance comparison with different topological features, In the Figure 5, we denote the “Hete+Diff,” “Hete,” and “Diff” as the Hetesim and diffusion combined feature, HeteSim feature, and diffusion feature, respectively. As we can see from Figure 5, the Hetesim and diffusion combined features achieved higher performance than the two separate features. The results show that the combination of the two features can improve the prediction performance. Figure 6 shows the ROC curve comparison with different feature groups, which is the method using GBDTL2E only with diffusion feature, using GBDTL2E only with HeteSim feature, and GBDTL2E with combined feature. We also used 10-fold cross validation to verify the influence of different feature groups on the experimental results. We can see, from Figure 6, that GBDTL2E with combined features can obtain higher performances than other two algorithms. The GBDTL2E with the Hetesim feature only could obtain a better performance than the GBDTL2E with the diffusion feature only.

### 3.5. Performance Comparison With Existing Methods

In this section, the GBDTL2E algorithm was compared with the existing methods for predicting associations between lncRNAs and EFs. However, there were a few studies that predicted new potential associations between lncRNAs and EFs. Three methods were chosen to compare with the proposed GBDTL2E method. These were KATZ (Vural and Kaya, 2018), MPALERLS (Xu, 2018), and BIRWAPALE (Xu, 2018).

- *KATZ*: The KATZ method, based on the KATZ, was used to find potential new associations between lncRNAs and EFs; it uses the DLREFD database as well and contains proven associations between lncRNAs and EFs. The KATZ and Gaussian interaction profile kernel similarity was used to predict new potential associations between lncRNAs and EFs. In this method, the parameters β and *k* are to 0.01 and 3, respectively.
- *MPALERLS*: The MPALERLS method used the Laplace operator for regularization, built the cost function and minimized it, and finally obtained the optimal classifier of lncRNAs space and EFs space. Finally, the two optimal classifiers were transformed into a unified classifier to calculate the probability matrix of lncRNA-EFs association relation. They used the classifier to calculate the probability of lncRNA-EFs association relation and to rank the lncRNA-EF association according to the probability score. We set the weight of lncRNAs classifier and EFs classifier to 0.4 and 3, respectively.
- *BIRWAPALE*: The BIRWAPALE method is a double random walk algorithm on heterogeneous networks. Finally, the double random walk converged in the heterogeneous network, and the probability score of lncRNAs and EFs association relationship could be obtained. The parameters α, *l*, and *r* are set to 1, 2, and 3.

**Figure 7:**
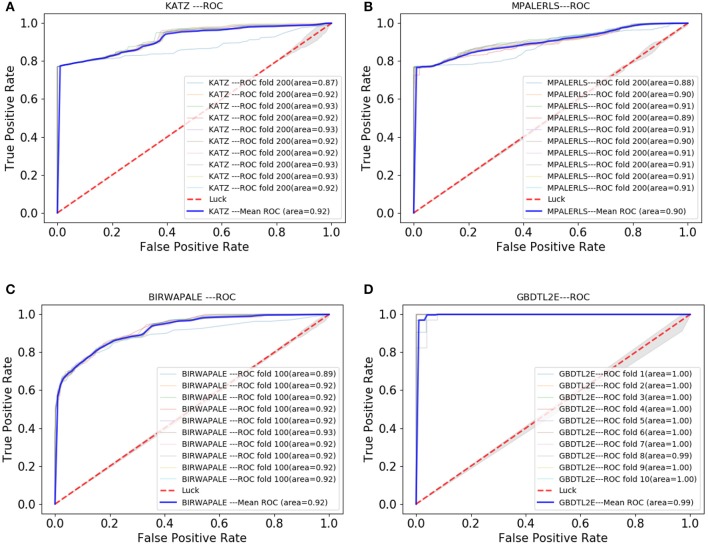
The Roc curve comparison with existing method. **(A)** The ROC curve only of KATZ. **(B)** The ROC curve only of MPALERLS. **(C)** The ROC curve of BIRWAPALE. **(D)** The ROC curve of GBDTL2E.

Figure 7 shows the comparison results. The experimental results show that the GBDTL2E algorithm can obtain a better performance than the other three algorithms. This was for several reasons: (1) Computing the HeteSim score of different paths from lncRNA to EFs in the heterogenous network to obtain the HeteSim features, and combining the HeteSim features and diffusion features as the data feature, could make better use of the topological characteristics of heterogeneous networks and thus obtain better performance. (2) The GBDT algorithm is an effective prediction model. As far as we know, we have been the first to apply both diffusion and HeteSim features to predict lncRNA-EFs interactions. As result show that, combine the diffusion and HeteSim features can further improve the performance.

### 3.6. Case Study

To further measure the performance of our proposed algorithm, we investigated an environmental factor “Cisplatin,” which is an effective chemotherapy drug for many cancers (Florea and Büsselberg, 2011). The proven associations between “Cisplatin” and many lncRNAs have been discovered. In this study, we attempted to use our model to predict the association between “Cisplatin” and lncRNA. First, all associations between “Cisplatin” and lncRNA were deleted from the training set.

After processed by our algorithm, we sorted the correlation values between “Cisplatin” and ordered LncRNA from largest to smallest. We found that all the top 10 lncRNAs were related to “Cisplatin,” and these lncRNAs are confirmed to be related to “Cisplatin” in the DLREFD database. The 10 lncRNAs and their corresponding PUBMED reference ID are shown in Table 4.

**Table 4:** The TOP 10 predicted lncRNAs related to cisplatin.

**Number**	**LncRNA name**	**PubMedID**
1	AK12669	23741487
2	AC015818.3	25250788
3	ABCC6P1	25250788
4	GABPB-AS1	24036268
5	CASC2	28495512
6	PSORS1C3	25250788
7	H19	28189050
8	AK125699	25250788
9	SRGAP3-AS2	25250788
10	XLOC_001406	25250788

## 4. Conclusions

Recent studies have shown that the interaction between lncRNA and EF is closely related to the production of diseases. As more and more computational methods are used to deal with biological problems, which can greatly save manpower, it is possible to use computational methods to predict the interaction between lncRNAs and EFs. In this paper, we proposed a method to predict the association between lncRNAs and EFs. The proposed method combined the Hetesim features and the diffusion features based on multi-feature fusion, and used the machine learning algorithm GBDT to predict the association between lncRNAs and EFs based on heterogeneous networks. The 10-fold cross validation was used to evaluate our method. We also compared our method with others. An environmental factor in the case study was also be used to compare our performance. The results show that the GDBTL2E can obtain high performance. In future, adding the expression profile of lncRNAs to further improve the performance will be investigated.

## Data Availability Statement

Publicly available datasets were analyzed in this study. This data can be found here: https://github.com/zhufangkuang/DLREFD.

## Author Contributions

JW, ZK, ZM, and GH conceived this work and designed the experiments. JW and ZK carried out the experiments. ZM and GH collected the data and analyzed the results. JW and ZK wrote, revised, and approved the manuscript.

### Conflict of Interest

The authors declare that the research was conducted in the absence of any commercial or financial relationships that could be construed as a potential conflict of interest.

## References

[B1] Bertone P. Stolc V. Royce T. E. Rozowsky J. S. Urban A. E. Zhu X.. (2004). Global identification of human transcribed sequences with genome tiling arrays. Science 306, 2242–2246. 10.1126/science.1103388 15539566

[B2] Burges C. J. (1998). A tutorial on support vector machines for pattern recognition. Data Min. Knowl. Discov. 2, 121–167. 10.1023/A:1009715923555

[B3] Caye K. Jumentier B. Lepeule J. François O. (2019). LFMM 2: fast and accurate inference of gene-environment associations in genome-wide studies. Mol. Biol. Evol. 36, 852–860. 10.1093/molbev/msz008 30657943 PMC6659841

[B4] Chen Q. Lai D. Lan W. Wu X. Chen B. Chen Y.-P. P.. (2020). ILDMSF: inferring associations between long non-coding RNA and disease based on multi-similarity fusion. IEEE ACM Trans. Comput. Biol. Bioinform. 10.1109/TCBB.2019.2936476 31443046

[B5] Chen X. (2016). miREFRWR: a novel disease-related microRNA-environmental factor interactions prediction method. Mol. Biosyst. 12, 624–633. 10.1039/C5MB00697J 26689259

[B6] Chen X. Liu M.-X. Cui Q.-H. Yan G.-Y. (2012). Prediction of disease-related interactions between microRNAs and environmental factors based on a semi-supervised classifier. PLoS ONE 7:e43425. 10.1371/journal.pone.0043425 22937049 PMC3427386

[B7] Chen X. Wang L. Qu J. Guan N.-N. Li J.-Q. (2018). Predicting miRNA-disease association based on inductive matrix completion. Bioinformatics 34, 4256–4265. 10.1093/bioinformatics/bty503 29939227

[B8] Chen X. Yan G.-Y. (2013). Novel human lncRNA-disease association inference based on lncRNA expression profiles. Bioinformatics 29, 2617–2624. 10.1093/bioinformatics/btt426 24002109

[B9] Cheng L. Qi C. Zhuang H. Fu T. Zhang X. (2020). gutmdisorder: a comprehensive database for dysbiosis of the gut microbiota in disorders and interventions. Nucleic Acids Res. 48, D554–D560. 10.1093/nar/gkz843 31584099 PMC6943049

[B10] Cho H. Berger B. Peng J. (2015). “Diffusion component analysis: unraveling functional topology in biological networks,” in International Conference on Research in Computational Molecular Biology, ed Przytycka T. M. (Cham: Springer International Publishing), 62–64. 10.1007/978-3-319-16706-0_9 PMC5524124 28748230

[B11] Cover T. Hart P. (1967). Nearest neighbor pattern classification. IEEE Trans. Inform. Theor. 13, 21–27. 10.1109/TIT.1967.1053964

[B12] Deng L. Li W. Zhang J. (2019a). LDAH2V: exploring meta-paths across multiple networks for lncRNA-disease association prediction. IEEE/ACM Trans. Comput. Biol. Bioinform. 10.1109/TCBB.2019.2946257 31725386

[B13] Deng L. Wang J. Xiao Y. Wang Z. Liu H. (2018). Accurate prediction of protein-lncRNA interactions by diffusion and HeteSim features across heterogeneous network. BMC Bioinformatics 19:370. 10.1186/s12859-018-2390-0 30309340 PMC6182872

[B14] Deng L. Wang J. Zhang J. (2019b). Predicting gene ontology function of human MicroRNAs by integrating multiple networks. Front. Genet. 10:3. 10.3389/fgene.2019.00003 30761178 PMC6361788

[B15] Ding L. Wang M. Sun D. Li A. (2018). TPGLDA: novel prediction of associations between lncRNAs and diseases via lncRNA-disease-gene tripartite graph. Sci. Rep. 8:1065. 10.1038/s41598-018-19357-3 29348552 PMC5773503

[B16] Eid A. Mhatre I. Richardson J. R. (2019). Gene-environment interactions in Alzheimer's disease: a potential path to precision medicine. Pharmacol. Ther. 199, 173–187. 10.1016/j.pharmthera.2019.03.005 30877021 PMC6827882

[B17] Florea A.-M. Büsselberg D. (2011). Cisplatin as an anti-tumor drug: cellular mechanisms of activity, drug resistance and induced side effects. Cancers 3, 1351–1371. 10.3390/cancers3011351 24212665 PMC3756417

[B18] Flynn R. A. Chang H. Y. (2014). Long noncoding RNAs in cell-fate programming and reprogramming. Cell Stem Cell 14, 752–761. 10.1016/j.stem.2014.05.014 24905165 PMC4120821

[B19] Friedman J. H. (2001). Greedy function approximation: a gradient boosting machine. Ann. Stat. 29, 1189–1232. 10.1214/aos/1013203451

[B20] Golub G. H. Reinsch C. (1971). “Singular value decomposition and least squares solutions,” in Linear Algebra (Berlin; Heidelberg: Springer), 134–151. 10.1007/978-3-662-39778-7_10

[B21] Gu C. Liao B. Li X. Cai L. Li Z. Li K.. (2017). Global network random walk for predicting potential human lncRNA-disease associations. Sci. Rep. 7:12442. 10.1038/s41598-017-12763-z 28963512 PMC5622075

[B22] Guo Z.-H. You Z.-H. Wang Y.-B. Yi H.-C. Chen Z.-H. (2019). A learning-based method for LncRNA-disease association identification combing similarity information and rotation forest. iScience 19, 786–795. 10.1016/j.isci.2019.08.030 31494494 PMC6733997

[B23] Hattori M. Tanaka N. Kanehisa M. Goto S. (2010). SIMCOMP/SUBCOMP: chemical structure search servers for network analyses. Nucleic Acids Res. 38(Suppl_2), W652–W656. 10.1093/nar/gkq367 20460463 PMC2896122

[B24] Huang Z. Shi J. Gao Y. Cui C. Zhang S. Li J.. (2018). HMDD v3.0: a database for experimentally supported human microRNA-disease associations. Nucleic Acids Res. 47, D1013–D1017. 10.1093/nar/gky1010 30364956 PMC6323994

[B25] Jiang Q. Wang Y. Hao Y. Juan L. Teng M. Zhang X.. (2008). miR2Disease: a manually curated database for microRNA deregulation in human disease. Nucleic Acids Res. 37(Suppl_1), D98–D104. 10.1093/nar/gkn714 18927107 PMC2686559

[B26] Lan W. Wang J. Li M. Liu J. Wu F.-X. Pan Y. (2018). Predicting microRNA-disease associations based on improved microRNA and disease similarities. IEEE ACM Trans. Comput. Biol. Bioinform. 15, 1774–1782. 10.1109/TCBB.2016.2586190 27392365

[B27] Li C. Liu H. Hu Q. Que J. Yao J. (2019). A novel computational model for predicting microRNA-disease associations based on heterogeneous graph convolutional networks. Cells 8:977. 10.3390/cells8090977 31455028 PMC6769654

[B28] Liaw A. Wiener M. (2002). Classification and regression by randomforest. R News 2/3, 18–22.

[B29] Liu H. Zhang W. Zou B. Wang J. Deng Y. Deng L. (2020). Drugcombdb: a comprehensive database of drug combinations toward the discovery of combinatorial therapy. Nucleic Acids Res. 48, D871–D881. 10.1093/nar/gkz1007 31665429 PMC7145671

[B30] Liu Y. Zeng X. He Z. Zou Q. (2016). Inferring microRNA-disease associations by random walk on a heterogeneous network with multiple data sources. IEEE ACM Trans. Comput. Biol. Bioinform. 14, 905–915. 10.1109/TCBB.2016.2550432 27076459

[B31] Luo H. Lan W. Chen Q. Wang Z. Liu Z. Yue X.. (2018). Inferring microRNA-environmental factor interactions based on multiple biological information fusion. Molecules 23, 2439. 10.3390/molecules23102439 30249984 PMC6222788

[B32] Luo Z. Jegga A. G. Bezerra J. A. (2018). Gene-disease associations identify a connectome with shared molecular pathways in human cholangiopathies. Hepatology 67, 676–689. 10.1002/hep.29504 28865156 PMC5834359

[B33] Moreau J. L. Kesteven S. Martin E. M. Lau K. S. Yam M. X. O'Reilly V. C.. (2019). Gene-environment interaction impacts on heart development and embryo survival. Development 146:dev172957. 10.1242/dev.172957 30787001

[B34] Opap K. Mulder N. (2017). Recent advances in predicting gene-disease associations. F1000Res. 6:578. 10.12688/f1000research.10788.1 28529714 PMC5414807

[B35] Ou-Yang L. Huang J. Zhang X.-F. Li Y.-R. Sun Y. He S.. (2019). LncRNA-disease association prediction using two-side sparse self-representation. Front. Genet. 10:476. 10.3389/fgene.2019.00476 31191605 PMC6546878

[B36] Ping P. Wang L. Kuang L. Ye S. Iqbal M. F. B. Pei T. (2018). A novel method for lncRNA-disease association prediction based on an lncRNA-disease association network. IEEE ACM Trans. Comput. Biol. Bioinform. 16, 688–693. 10.1109/TCBB.2018.2827373 29993639

[B37] Qiu C. Chen G. Cui Q. (2012). Towards the understanding of microRNA and environmental factor interactions and their relationships to human diseases. Sci. Rep. 2:318. 10.1038/srep00318 22428086 PMC3306023

[B38] Shi C. Kong X. Huang Y. Philip S. Y. Wu B. (2014). Hetesim: A general framework for relevance measure in heterogeneous networks. IEEE Trans. Knowl. Data Eng., 26, 2479–2492. 10.1109/TKDE.2013.2297920

[B39] Sun Y.-Z. Zhang D.-H. Ming Z. Li J.-Q. Chen X. (2017). DLREFD: a database providing associations of long non-coding RNAs, environmental factors and phenotypes. Database 2017:bax084. 10.1093/database/bax084 29220470 PMC5737057

[B40] Tang C. Zhou H. Zheng X. Zhang Y. Sha X. (2019). Dual laplacian regularized matrix completion for microRNA-disease associations prediction. RNA Biol. 16, 601–611. 10.1080/15476286.2019.1570811 30676207 PMC6546388

[B41] Vural H. Kaya M. (2018). Prediction of new potential associations between LncRNAs and environmental factors based on KATZ measure. Comput. Biol. Med. 102, 120–125. 10.1016/j.compbiomed.2018.09.019 30268976

[B42] Xu Y. Wu M. Zhang Q. Ma S. (2019). Robust identification of gene-environment interactions for prognosis using a quantile partial correlation approach. Genomics 111, 1115–1123. 10.1016/j.ygeno.2018.07.006 30009922 PMC6335188

[B43] Xu Z. (2018). Prediction of correlation between long non-coding RNA and environmental factors based on nuclear similarity of gaussian interaction attributes (Master's thesis). South China University of Technology, Guangzhou, China.

[B44] Yan C. Wang J. Ni P. Lan W. Wu F.-X. Pan Y. (2017). DNRLMF-MDA: predicting microRNA-disease associations based on similarities of microRNAs and diseases. IEEE ACM Trans. Comput. Biol.Bioinform. 16, 233–243. 10.1109/TCBB.2017.2776101 29990253

[B45] Yu G. Fu G. Lu C. Ren Y. Wang J. (2017). BRWLDA: bi-random walks for predicting lncRNA-disease associations. Oncotarget 8:60429. 10.18632/oncotarget.19588 28947982 PMC5601150

[B46] Zhang J. Zhang Z. Chen Z. Deng L. (2019). Integrating multiple heterogeneous networks for novel lncRNA-disease association inference. IEEE ACM Trans. Comput. Biol.Bioinform. 16, 396–406. 10.1109/TCBB.2017.2701379 28489543

[B47] Zhang Z. Zhang J. Fan C. Tang Y. Deng L. (2019). KATZLGO: large-scale prediction of LncRNA functions by using the KATZ measure based on multiple networks. IEEE ACM Trans. Comput. Biol.Bioinform. 16, 407–416. 10.1109/TCBB.2017.2704587 28534780

[B48] Zhou J. Shi Y.-Y. (2018). A bipartite network and resource transfer-based approach to infer lncRNA-environmental factor associations. IEEE ACM Trans. Comput. Biol.Bioinform. 15, 753–759. 10.1109/TCBB.2017.2695187 28436883

[B49] Zhou M. Han L. Zhang J. Hao D. Cai Y. Wang Z.. (2014). A computational frame and resource for understanding the lncRNA-environmental factor associations and prediction of environmental factors implicated in diseases. Mol. Biosyst. 10, 3264–3271. 10.1039/C4MB00339J 25308527

